# Effect of Supplemental Essential Oils Blend on Broiler Meat Quality, Fatty Acid Profile, and Lipid Quality

**DOI:** 10.3390/ani15070929

**Published:** 2025-03-24

**Authors:** Mohamed Kahiel, Kai Wang, Haocong Xu, Jian Du, Sheng Li, Dan Shen, Chunmei Li

**Affiliations:** Research Centre for Livestock Environmental Control and Smart Production, College of Animal Science and Technology, Nanjing Agricultural University, Nanjing 210095, China; mohammed.kaheil@agr.menofia.edu.eg (M.K.); wk@stu.njau.edu.cn (K.W.); xhc2000a@163.com (H.X.); dujian@stu.njau.edu.cn (J.D.); lisheng9395@163.com (S.L.)

**Keywords:** essential oils, growth performance, meat quality, lipid metabolism, gene expression

## Abstract

This investigation evaluates the impact of the EOB on chicken growth performance, meat quality, and lipid metabolism. Two hundred and fifty-six one-day-old, white-feathered broilers were randomly allocated to four groups. Each group was subdivided into eight replicates, each with eight unsexed chicks, including the control group (CON), EOB_150_, EOB_250_, and EOB_350_, with 0, 150, 250, and 350 mg/L of the EOB added to the drinking water, respectively. The expression levels of genes associated with antioxidants and lipid metabolism were analyzed using real-time polymerase chain reaction (RT-PCR). Additionally, the FA profile of the breast muscle was determined using gas chromatography. The data displayed that those birds in the EOB_250_ group had a higher breast muscle index compared to the CON group. The breast meat in the EOB groups showed that there is increased yellowness, water holding capacity (WHC), and polyunsaturated fatty acids (PUFAs), while cooking losses, drip losses, and saturated fatty acids (SFAs) were reduced compared to the CON. The application of supplements for the EOB_250_ and EOB_350_ groups increased antioxidant indices as well as the expression of antioxidant-related genes in the liver and muscles. However, these groups decreased the concentrations of triglycerides (TG), total cholesterol (TC), and low-density lipoprotein (LDL-C) in serum and liver compared to the EOB_150_ and CON groups. These EOB groups downregulated expression of some genes linked to liver FA synthesis and elevated the expressions of lipid β-oxidation-related genes compared to the CON. It can be concluded that the supplementation with 250 mg/L of the EOB has the potential as an alternative water additive in the broiler industry.

## Simple Summary

Essential oils (EOs) are regarded as a promising alternative to antibiotics, owing to their growth-promoting and antioxidant properties. However, studies on the inclusion of EOs in drinking water (DW) and their effects on meat quality remain limited. Therefore, this study aims to explore the impact of the essential oils blend (EOB) supplementation in the broiler DW on performance, meat quality, fatty acid (FA) profile, and the expression of genes related to lipid metabolism. The findings demonstrate that the EOB supplementation can improve meat quality by stabilizing antioxidants, enhancing the lipid quality index, promoting fatty acid oxidation, and suppressing the activity of genes associated with lipid metabolism, particularly those involved in fat synthesis.

## 1. Introduction

The global demand for inexpensive and highly palatable protein has driven the tremendous growth of the poultry sector [1]. For decades, antibiotics were administered to promote growth and to protect chickens against pathogenic microbes [2]. Antibiotics are banned in the European Union and China because of their unwanted effects, including antibiotic-resistant bacteria, residue contamination of animal products, and ecosystem contamination [3]. The replacement of synthetic growth enhancers and antibiotics with organic nutritional supplements was reported to improve the birds’ immunity, growth, and carcass quality [4,5]. EOs have emerged as a potential substitute for antibiotics in broiler production because of their natural antibacterial and anti-inflammatory characteristics. Thus, utilizing these plant-extracted compounds can enhance performance and gut health, as well as meat quality and oxidative stability, without the risks associated with antibiotic residue and antibiotic-resistant bacteria proliferation [6].

One of the natural strategies that has been widely used in broiler production is using oregano essential oil (OEO), which has contributed to positive results in several studies [7]. These studies indicate that OEO acts as a growth promoter, a natural antibiotic, and acts to elevate beneficial bacteria in the digestive tract [8,9]. OEO has also been suggested as an efficient mechanism for enhancing broiler meat quality [10]. Carvacrol and thymol are principal volatile compounds present in OEO, which contribute to the biological activity of the oil. Orange EO contains a plethora of compounds and is composed of 85–99% volatile constituents, which are extracted from the peel of the fruit [11]. Most of these volatile compounds are terpenoids and their oxygenated derivatives. EOs of orange and thyme were reported to also inhibit lipid oxidation in meat without compromising quality [12]. Cinnamon spice is achieved from the inner surface of *Cinnamomum verum*, a hardy, evergreen plant that is part of the Lauraceae family and has natural aromatic properties. Cinnamon and its EOs and bioactive compounds, including cinnamaldehyde and eugenol, are commonly used in poultry production as dietary additives. Cinnamon supplementation has been found to enhance meat quality and improve its shelf life [13].

Many researchers have observed the influence of EOs by adding them to broiler feeds and studying their impact on performance and meat quality data [7,14]. Another approach is to include EOs in DW to observe how EOs affect broiler performance. The EOB supplementation has been shown to increase the carcass yield, tenderness, and WHC in chickens [15]. EOs are reported to have anti-oxidative properties on lipids in tissues and serum, meanwhile enhancing meat quality and extending shelf life [16]. Based on previous studies, EOs in DW have the potential to enhance the growth, health, and meat quality of birds. EO supplementation has demonstrated improvements in productivity and antioxidant capacity in chickens [17]. Lipid peroxidation (MDA) is a common indication of oxidative stress in poultry [18], suggesting that EOs may reduce MDA levels, improve health tissues, and thus improve meat quality. Chickens that were administered EOs have also reported decreased TC, TG, and LDL-C [19], indicating that EOs may contribute to lipid metabolism regulation. Lipid content is closely related to metabolic disorders, like atherosclerosis, coronary heart disease, and fatty liver. Both total lipid intake and the ratio of FAs in the daily diet, when properly managed, can reduce the incidence of cardiovascular disease [20]. Moreover, lipid oxidation could also influence the quality and flavor of meat during FA production [21]. As an example, chicken breast meat, which has high levels of unsaturated fatty acids (UFAs), can decrease both the thrombogenicity and atherogenicity indices [22]. Oregano oil and cinnamon oil have been shown to have antibacterial and growth-promoting effects, and are widely used in poultry production. In addition, limonene has also been shown to improve antioxidant properties and gut health in broilers [23].

Few studies have been conducted to investigate whether the EOB extract supplementation from oregano leaves, cinnamon, and orange peels in broiler DW affects their meat quality and lipid metabolism. Thus, the purpose of this experiment is to thoroughly assess the influence of the EOB supplements in the DW on growth performance, breast meat quality, oxidative functions, FA profiles, and lipid quality indexes. This study applies valuable insight into broiler production.

## 2. Materials and Methods

### 2.1. Source of the EOB

The EOB used in the current study is a commercial product provided by Yangzhou Well Biological Technology Co., Ltd. (Nanjing, China). According to Zhang et al. [24], the EOB was extracted via steam distillation from the leaves of oregano, the peels of cinnamon, and the peels of oranges, and they are mixed in equal proportions. Due to the hydrophobic nature of the EOs, Tween-20 was incorporated as an emulsifier to enhance the solubility and stability in water. The final mixture consists of 10% essential oils (EOs), 50% Tween-20 emulsifier, and 40% pure water.

### 2.2. The Trial Design, Birds, and Treatments

A total of 256 one-day-old, unsexed AA broiler chicks (46.30 ± 0.23 g) were purchased from a broiler breeder company in Nanjing, China. Using a completely randomized design, it was randomly distributed to 4 groups of 8 replicates, each with 8 chicks/replicate. The experimental treatments included the CON, EOB_150_, EOB_250_, and EOB_350_, supplemented with EOB in DW (0, 150, 250, and 350 mg/L). The EOB supplementation levels were determined based on established efficacious concentrations (100–500 mg/kg or mg/L) of oregano, cinnamon, and orange peel essential oils in poultry nutrition, as demonstrated in the previous studies [19,25,26,27]. Optimal dosage was adjusted according to bioactive compound composition and corresponding biological activities. The EOB was fully dissolved in DW to ensure homogeneous distribution and was provided continuously throughout the trial. The trial phase continued for 42 d, involving the early phase (1 to 21 D) and end phase (22 to 40 D). The room temperature was maintained at 34–35 °C for the first day and decreased until the temperature was 21–22 °C. The experimental rations were similar across each group and prepared by NRC (1994). Table 1 displays the basic formula and nutritional level. Birds received ad libitum feed and water in galvanized wired cages measuring (90 cm × 60 cm × 35 cm). During early growth, each group (64 chicks) was housed in 8 cages (8 birds/cage). As the birds matured, housing density was reduced by doubling cage numbers to 16 (2 per replicate), while maintaining the original dimensions, ensuring adequate space for welfare and development. Upon arrival, the chicks were subjected to a continuous illumination regimen featuring a lighting routine of 23 h of light and 1 h of dark.

### 2.3. Estimation of Growth Performance and Carcass Traits

During the 42-day experimental period, total body weight and remaining feed per pen were measured and recorded on days 1, 21, and 42, in the early morning following an 8-h feed deprivation. Average body weight (ABW), average daily gain (ADG), average daily feed intake (ADFI), and feed conversion ratio (FCR) were calculated. Mortality was monitored daily, and deceased birds were recorded and weighed to adjust growth performance parameters, including gain, feed intake, and FCR estimates as appropriate.

At day 42 of the experiment, eight birds from each treatment group were randomly selected based on the average body weight and humanely slaughtered by cervical dislocation following a 12 h fasting period to evaluate slaughter performance. The birds were slaughtered in a designated slaughterhouse next to the poultry farm to avoid the stress of transportation. The body weight of all birds was measured before slaughter. After euthanasia, the birds were scalded and processed, and feathers, heads, viscera, and feet were removed. Post-evisceration, the semi-eviscerated carcass, fully eviscerated carcass, abdominal fat, breast muscle, liver, spleen, and Fabricius bursa were anatomically dissected and weighed alone. These components were weighed and calculated as a proportion relative to the bird’s body weight.

### 2.4. Sampling

At day 42, eight birds from every treatment group were chosen and weighed. Blood samples were obtained from the wing vein and transfused into a coagulation tube. The serum was isolated by centrifugation (3000 rpm for 15 min, 4 °C) and kept at −20 °C, and the birds were slaughtered through cervical dislocation for tissue samples. Parameters of meat quality were measured in the breast muscles. Subsequently, the liver and breast muscles were isolated and weighed, then rapidly collected, immediately frozen in liquid nitrogen, and kept at −80 °C for further measurement.

### 2.5. Biochemical Analysis

Blood samples were obtained from the wing vein of eight chickens of each group at 42 days old. The contents of TG, TC, LDL-C, and HDL-C in serum and liver were evaluated utilizing specific commercial kits, as per the producer’s directions (Nanjing Jiancheng Biotechnology Co., Ltd., Nanjing, China).

### 2.6. Meat Quality Parameters Assess

Meat samples were collected from eight chickens per group for physicochemical analysis. The pH of the breast muscle was measured 45-min post-slaughter using a pH meter (HI9125, Hanna Instruments, Cluj-Napoca, Romania). Three measurements were taken per sample, and the mean value was recorded. Meat color parameters, including lightness (L*), redness (a*), and yellowness (b*), were evaluated one hour post-slaughter using a chromameter (CR-410, Konica Minolta, Tokyo, Japan). To ensure accurate measurements, meat samples had a minimum thickness of 1.5 cm. Additionally, total color change (ΔE), chroma (saturation index), hue angle, and browning index (BI) were calculated using established equations as described in [28]. Color assessments were performed at three distinct locations on the left pectoralis minor muscle, and the mean value was used for analysis.

Water holding capacity was estimated using the technique described in [29], with some modifications. Breast fillets were sampled by the cranial side after 24 h exposure and cut into approximately 10 g cubes. Both samples were double analyzed. They were sandwiched between two pieces of filter paper and pressed a weight of 10 kg for 5 min. The weight of the samples was recorded before and after compression, and WHC was quantified based on the amount of exuded water. The following equation was used to calculate WHC:

WHC = 100 − {[(initial weight − final weight)/initial weight] × 100}.(1)

Drip loss was measured based on a method described in [13]. The breast portions utilized for the drip test were individually weighed, vacuum packed, and stored at 4 °C for 24 h. The weight change before and after storage was determined and calculated as a proportion of drop loss.

For cooking loss, approximately 30 g of meat samples were precisely cut (2 cm × 2 cm × 5 cm) and weighed in a plastic sack. Samples were heated in a thermostat water bath maintained at 85 °C, sufficient to raise the internal temperature of the meat to 77 °C, cooled, and reweighed. Cooking loss was determined based on the following formula:

The cooking loss percentage = [(initial weight − cooked weight)/initial weight] × 100(2)

Shear force (SF) has determined the same samples that were used to measure cooking loss. According to [30] protocol, we obtained two adjacent columns of 2.5 cm^2^ from each meat sample that were used to measure cooking loss. Using a shear device (C-LM3, Northeast Agricultural University, Harbin, China), each column was cut three times crosswise with surface muscle fibers being oriented in a longitudinal direction. We subsequently measured maximum SF (N) from each cutting. The two columns were averaged, and mean values were used to calculate the shear value for each sample.

#### Meat Texture Analysis

Meat samples taken from 2 cylinders (diameter: 20 mm, height: 20 mm) were sliced perpendicular to muscle fiber direction and subjected to textural profile determination using a texture analyzer (XT Plus, Stable Micro Systems Ltd., Godalming, UK) based on [31]. Samples were subjected to a set of parameters: 2 compressions with a compression ratio of 50%, a pre-test rate of 5 mm/s, a test rate of 5 mm/s, a post-test rate of 10 mm/s, a actuate pressure of 5 g, and a test duration of 1 s [32].

### 2.7. Antioxidant Capacity Analysis

The activities of antioxidant enzymes [total superoxide dismutase (T-SOD; A001-1), glutathione peroxidase (GSH-Px; A005-1), catalase (CAT; A007-1), total antioxidant capacity (T-AOC; A015-1) and MDA (MDA; A003-1)] in serum, liver, and breast muscle samples were assayed by commercial analytical kits according to the manufacturer’s recommendations (Jiancheng Bioengineering Institute, Nanjing, China). Total protein content was assessed using a bicinchoninic acid assay (P0009, Beyotime, Shanghai, China).

### 2.8. Fatty Acid Profile Determination

In breast muscle samples, lipids were isolated using chloroform–methanol following the protocol in [33]. Gas chromatographic analysis was performed on sodium hydroxide/methanol-FA-methyl esters. Fatty acyl compositions were analyzed with an SP-2380 capillary chromatographic column (100 m × 0.25 mm × 0.20 µm; ANPEL Laboratory Technologies Inc., Shanghai, China) using a 7890A gas chromatograph (Agilent Technologies Co., Ltd., Palo Alto, CA, USA) with a flame ionization sensor for fatty acyl methyl esters. The FA was identified by comparing the stay periods to those of the established reference substances obtained from ANPEL Laboratory Technologies Inc. (Shanghai, China). The composition of FAs was measured using internal standard method [34].

#### Lipid Quality Indexes

Different lipid parameters were calculated by comparing ratios, including n-6/n-3 PUFA, ΣPUFA/ΣSFA, linoleic acid/α-linolenic acid (LA/ALA), eicosapentaenoic acid and docosahexaenoic acid (EPA + DHA), unsaturation index (UI), health-promoting index (HPI), flesh lipid quality (FLQ), nutrition value index (NVI), Index of Atherogenicity (IA), thrombosis index (TI), hypocholesterolemic/hypercholesterolemic (h/H), peroxidation trend index (PI), inflammatory biomarker (IB), dietary fatty acids (DFAs), nutritional ratio (NR), thioesterase, elongase, and desaturase indices, of broiler muscle utilizing equations according to [35,36].

### 2.9. RNA Extraction and RT-qPCR Verification

Total RNA was attained by TRIzol™ Total RNA Kit (Vazyme Biotech Co., Ltd., Nanjing, China). The quantity of RNA was assessed using Nanodrop 2000 (Thermo Fisher Scientific, Wilmington, DE, USA), and cDNA reverse transcription was carried out using ABScript III RT Master Mix for qPCR with gDNA Remover (ABclonal, Wuhan, China). According to instructions of SYBR Green I (ABclonal, Wuhan, China) real-time fluorescence quantitative PCR kit, the total reaction system was 20 μL, and reagents required for the reaction were applied as needed. After this, the melting curve was run and analyzed to check whether the process was acceptable. The mRNA expression of the target gene was normalized by β-actin (∆CT), and the PCR data were analyzed by the 2^−∆∆CT^ method. RT-qPCR was performed to measure expression levels of genes encoding antioxidant mediators in the liver and muscles (CAT, SOD, GSH, and nuclear factor erythroid 2-related factor 2 (NRF2)), and genes related to lipid metabolism [sterol regulatory element-binding protein-1c (SREBP-1c), acetyl-CoA carboxylase (ACC), FA synthase (FAS), FA desaturase-2 (FADS2), stearoyl-CoA desaturase (SCD), peroxisome proliferator activated receptor gamma (PPARγ), CCAAT / Enhancer Binding Protein α (C/EBPα), peroxisome proliferator-activated receptor alpha (PPARα), acyl-CoA oxidase 1 (ACOX1), carnitine palmitoyl transferase 1a (CPT1), lipoprotein lipase (LPL), FA-binding protein (FABP), and FA transport protein 1 (FATP1)]. The RT-qPCR assay primer sequences are provided in Table 2.

### 2.10. Statistical Analysis

Statistical analysis for each data group was carried out by SPSS 27.0 software. Group differences were evaluated via one-way ANOVA, and individual comparisons were measured by the Duncan test. Graphs were made using GraphPad Prism 9.0 software (GraphPad Software, San Diego, CA, USA). Data are expressed as an average ± SEM; *p* < 0.05 is indicated for significance assertion.

## 3. Results

### 3.1. Growth Performance

Supplementation with the EOB resulted in variable growth performance at different doses in the broilers. As indicated in Table 3, no differences were observed in the ABW, ADG, ADFI, and FCR through the 1–21 d period. During the overall 1–42 d experimental period, the EOB_250_ group demonstrated an increased ABW and reduced FCR, but there were no variations between the groups (*p* > 0.05).

### 3.2. Carcass Traits

As illustrated in Table 4, the breast muscle index percentage of the EOB_250_ group was significantly greater than the CON (*p* < 0.05). But there were no differences observed on carcass traits, such as the liver index, spleen index, bursa of Fabricius index, abdominal fat index, semi-evisceration index, and full-evisceration index among the four groups (*p* > 0.05).

### 3.3. Biochemical Indices in Serum and Liver

**Figure 1:**
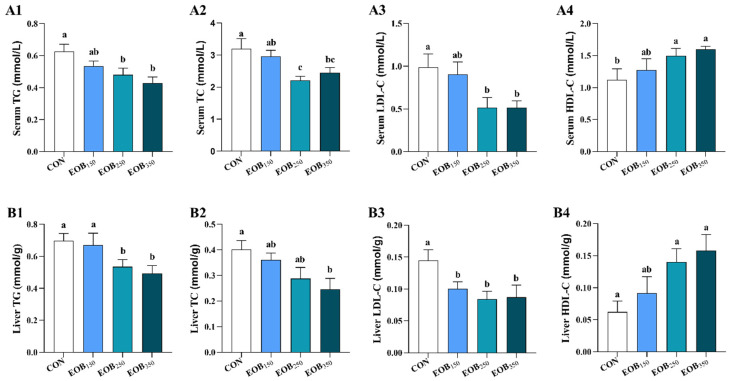
Effects of essential oils blend on biochemical indices in the serum and liver of broilers. Results are expressed as means ± standard error of the mean (SEM). ^a–b^ Mean values above the column carrying various letters vary statistically (*p* < 0.05). (**A1**–**A4**) the serum biochemical indices and (**B1**–**B4**) the liver biochemical indices TG: triglyceride; TC: total cholesterol; LDL-C: low-density lipoprotein-cholesterol; HDL-C: high-density lipoprotein cholesterol. CON = control drinking water (without EOB); EOB_150_ = DW + 150 mg/L EOB; EOB_250_ = DW + 250 mg/L EOB; EOB_350_ = DW + 350 mg/L EOB.

The impacts of the EOB supplementation on lipid metabolism in chickens are displayed in Figure 1. Compared to the CON group, the EOB_250_ and EOB_350_ supplementation decreased TG and LDL-C levels, while HDL-C levels were enhanced in both the liver and serum (*p* < 0.05). Moreover, the EOB_250_ supplementation also reduced the serum TC levels (*p* < 0.05), while the EOB_350_ decreased the liver TC levels relative to the CON (*p* < 0.05).

### 3.4. Meat Quality

The impact of the EOB supplementation on meat quality is shown in Table 5. No differences in the post-slaughter pH values were found among the treatment groups. Nevertheless, other quality parameters improved substantially. Meat from groups supplemented with the EOB showed significantly greater WHC in breast meat (*p* < 0.05). Furthermore, the EOB_250_ and EOB_350_ groups had significantly less drip loss (*p* < 0.05). Cooking loss was also reduced by the EOB supplementation, with the lowest values from the EOB_350_ group (*p* < 0.05). Moreover, notable differences in meat color parameters (b*, chroma, and BI) were observed. These parameters in the EOB_250_ group were higher than those in the EOB_150_ and CON groups (*p* < 0.05). By contrast, no effects were observed on L*, a*, ΔE, or hue angle (*p* > 0.05).

#### Meat Texture Analysis

**Figure 2:**
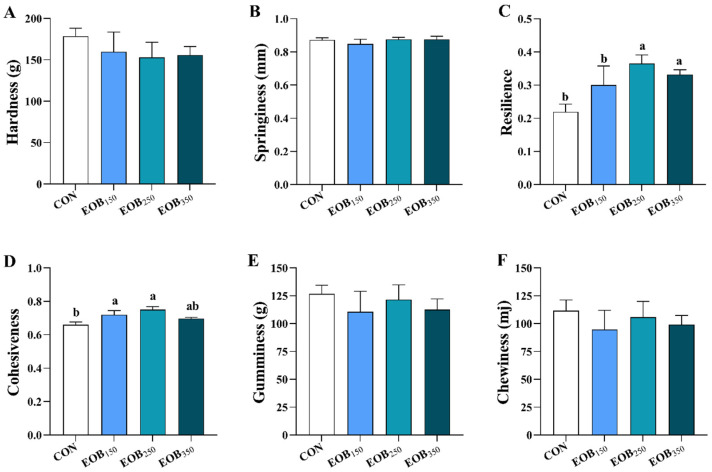
Effects of essential oils blend on meat texture analysis of chicken breast muscle. Results are expressed as means ± standard error of the mean (SEM). ^a,b^ Mean values above the column carrying various letters vary statistically (*p* < 0.05). (**A**) Hardness, (**B**) Springiness, (**C**) Resilience, (**D**) Cohesiveness, (**E**) Gumminess, And (**F**) Chewiness CON = control drinking water (without EOB); EOB_150_ = DW + 150 mg/L EOB; EOB_250_ = DW + 250 mg/L EOB; EOB_350_ = DW + 350 mg/L EOB.

The texture analysis of the breast meat is illustrated in Figure 2. The EOB_250_ and EOB_350_ groups exhibited higher resilience values than the EOB_150_ and CON (*p* < 0.05). Moreover, the CON group displayed a lower meat cohesiveness value than the EOB_150_ and EOB_250_ (*p* < 0.05). No variations were found in other parameters of texture; however, hardness, gumminess, chewiness, and springiness were numerically higher in the CON group (*p* > 0.05).

### 3.5. Antioxidant Activity

**Figure 3:**
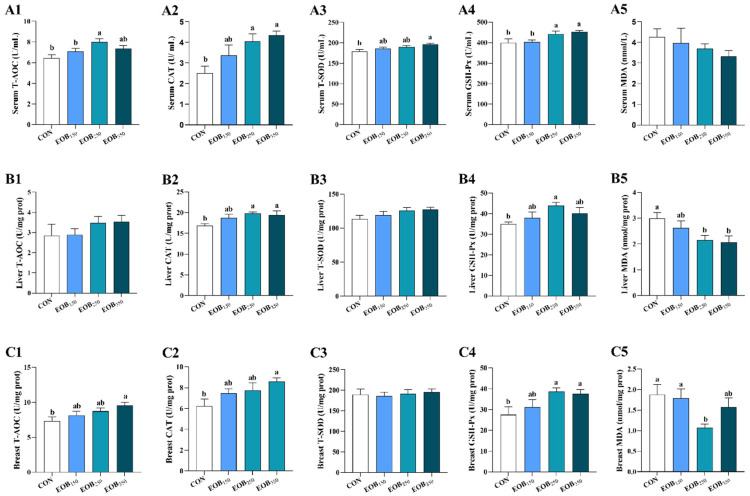
Effects of essential oils blend on the antioxidant capacity of broilers. Results are expressed as means ± standard error of the mean (SEM). ^a,b^ Mean values above the column carrying various letters vary statistically (*p* < 0.05). (**A1**–**A5**) Effect of the EOB on serum antioxidant enzyme activities. (**B1**–**B5**) Effect of the EOB on liver antioxidant enzyme activities. (**C1**–**C5**) Effect of the EOB on antioxidant enzyme activities. Abbreviations: T-AOC: total antioxidant capacity; CAT: catalase; T-SOD: total superoxide dismutase; GSH-Px: glutathione peroxidase; MDA: malondialdehyde. CON = control drinking water (without EOB); EOB_150_ = DW + 150 mg/L EOB; EOB_250_ = DW + 250 mg/L EOB; EOB_350_ = DW + 350 mg/L EOB.

The results showed that the EOB supplementation increased antioxidant activity in the serum (Figure 3, A1–A5). T-AOC was raised in the EOB_250_ group relative to both the EOB_150_ and CON. Moreover, the EOB_350_ group showed increased T-SOD activity compared to the CON (*p* < 0.05). Both CAT and GSH-Px activities were higher in the EOB_250_ and EOB_350_ groups, in contrast to the CON (*p* < 0.05). There were no changes between the groups for MDA levels (*p* > 0.05).

The activity of antioxidant enzymes in the liver (B1–B5) and breast muscle (C1–C5) is displayed in Figure 3. In the liver, relative to the CON group, CAT activity was higher in both the EOB_250_ and EOB_350_ (*p* < 0.05). Moreover, GSH-Px activity in the EOB_250_ group was noticeably higher in comparison with the CON (*p* < 0.05). Furthermore, MDA levels were lower with augmenting amounts of the EOB groups (*p* < 0.05). In the breast muscle, the EOB_350_ supplementation markedly elevated both CAT and T-AOC levels (*p* < 0.05). Moreover, the activity of GSH-Px was amplified in both the EOB_250_ and EOB_350_ groups, relative to the CON (*p* < 0.05).

### 3.6. Hepatic and Breast Muscle Antioxidant-Related Gene Expression

**Figure 4:**
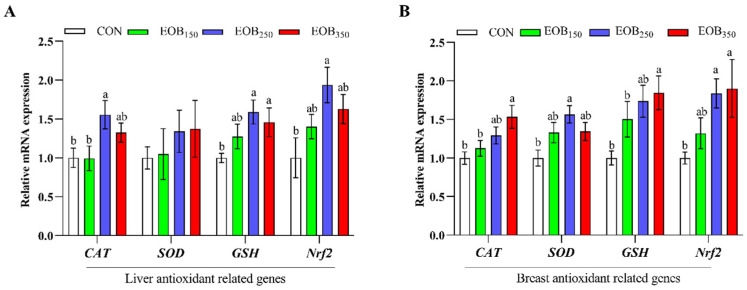
The mRNA expression of antioxidant-related genes in the liver and breast muscle of the broilers. Results are expressed as means ± standard error of the mean (SEM). ^a,b^ Mean values above column carrying various letters vary statistically (*p* < 0.05). (**A**) liver antioxidant-related genes and (**B**) breast antioxidant-related genes. CON = control drinking water (without EOB); EOB_150_ = DW + 150 mg/L EOB; EOB_250_ = DW + 250 mg/L EOB; EOB_350_ = DW + 350 mg/L EOB.

Figure 4 shows the effect of the EOB supplementation on the regulation of antioxidant-related genes in the liver and breast muscle. In the liver, mRNA expression levels of *CAT* and *NRF2* were elevated in the EOB_250_ group compared with the EOB_150_ and CON (*p* < 0.05). Likewise, expression of the *GSH* gene was enhanced in the EOB_250_ and EOB_350_ groups, in comparison to the CON (*p* < 0.05). In the breast muscle, supplementation with EOB_350_ markedly upregulated mRNA levels for *CAT* and *GSH* (*p* < 0.05). Throughout, the expression of the *SOD* gene was upregulated in the EOB_250_ group relative to the CON (*p* < 0.05). Moreover, the expression level of the *NRF2* gene was amplified in the EOB_250_ and EOB_350_ groups compared to the CON (*p* < 0.05).

### 3.7. Fatty Acid Profile and Lipid Quality Indexes

Table 6 presents the influence of the EOB supplementation on the FA profile of the breast muscle. The results indicate that supplementation with EOB_250_ and EOB_350_ reduced levels of SFAs, such as C18:0 and C12:0 (*p* < 0.05). Contradictorily, the concentrations of PUFAs, particularly C18:2n6c, C18:3n3, C20:4n6, and C22:4n6, were higher in the EOB_250_ and EOB_350_ groups in comparison to the CON. The level of monounsaturated fatty acids (MUFAs) was greater in the EOB_250_ group than in the EOB_150_ and CON, although the difference was not significant (*p* > 0.05). Furthermore, supplementation with EOB_250_ and EOB_350_ increased concentrations of n-3 and n-6 PUFAs in chicken breast meat (*p* < 0.05).

Table 7 indicates a notable rise (*p* < 0.05) in the PUFA/SFA ratio, as well as in EPA + DHA, UI, HPI, h/H, FLQ, PI, and DFA in the EOB_250_ and EOB_350_ groups compared to the EOB_150_ and CON. Conversely, a significant decrease was observed in the AI, TI, and NR in the EOB_250_ and EOB_350_ groups (*p* < 0.05). Furthermore, the activities of Δ9-desaturase (18) and Δ9-desaturase (16 + 18) were higher in the EOB_250_ and EOB_350_ group compared to the CON (*p* < 0.05). Nevertheless, no variations were detected in the activities of elongase, thioesterase, Δ9-desaturase (16), and activity index across the treatment groups (*p* > 0.05).

### 3.8. Gene Expression Related to Hepatic Lipid Metabolism

**Figure 5:**
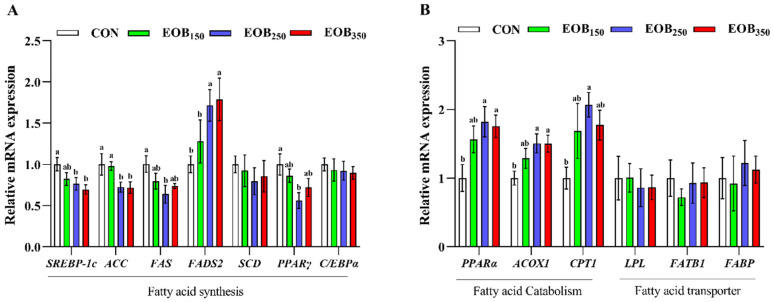
The effect of essential oils blend on the mRNA expression levels of liver lipid metabolism-related genes in broilers. Results are expressed as means ± standard error of the mean (SEM). ^a,b^ Mean values above the column carrying various letters vary statistically (*p* < 0.05). (**A**) fatty acid synthesis and (**B**) fatty acid for catabolism and transporter. CON = control drinking water (without EOB); EOB_150_ = DW + 150 mg/L EOB; EOB_250_ = DW + 250 mg/L EOB; EOB_350_ = DW + 350 mg/L EOB.

The effect of the EOB supplementation on lipid metabolism was assessed in the liver by measuring the mRNA expression of important FA biosynthesis enzymes (Figure 5A). The EOB_250_ and EOB_350_ group showed downregulated mRNA expression of *SREBP-1c* and *ACC* (*p* < 0·05); while *FADS2* expression was notably increased in the EOB_250_ and EOB_350_ groups compared with the EOB_150_ and CON. Moreover, the EOB_250_ group displayed significantly fewer expressions of *FAS* and *PPARγ* genes relative to the CON (*p* < 0·05). Further analysis announced that genes related to FA catabolism, including *PPARα*, *ACOX1*, and *CPT1*, had higher expression levels in the EOB_250_ group relative to the CON (*p* < 0·05), as shown in Figure 5B. However, no changes were observed in the expression of *FATB1*, *C/EBPα*, *LPL*, *SCD*, or *FABP* across the different treatment groups (*p* > 0.05).

## 4. Discussion

The present study describes the impacts of varying levels of EOB supplementation on growth attributes, carcass traits, meat FA composition, and oxidative stability. The results of this study showed that there were no significant differences in growth performance between the treatments throughout the experimental period. Conversely, along with our findings, ref. [37] observed higher chicken profitability and similar growth efficiency measures across all dietary regimens when essential oil blends were included as feed additives. The antioxidant properties of EO supplements, along with the regulation of digestive enzyme activity, may support intestinal function and consequently higher nutrient utilization, leading to superior growth rates [38]. Earlier research has discussed similar results with no negative effects on ADFI or BW that were observed for Ross broilers that received DW supplemented with a total of two types of Mexican OEO at 400 mg/L [27]. Variability in EO dosage, plant source, chemical composition, oil type, and environment could account for these differences in experimental findings.

Slaughter characteristics are an important performance indicator of meat production for both poultry and livestock, and are directly related to the profit of the chicken industry. The breast muscle index in the EOB_250_ group showed an increase compared to the CON group. This result is consistent with {Formatting Citation}, who found that a mixture of EO supplementation increased the carcass yield of birds, while other slaughter characteristics were not affected among treatments. EOs include carvacrol, which promotes pancreatic secretions, increasing digestive efficiency and the assimilation of nutrients. This process ultimately leads to higher carcass production [39]. The researchers believed an increase in valuable muscle mass was caused by EOs’ support of muscle development. However, other research has shown that EOs and organic acid supplementation did not affect the carcass characteristics in chickens [40]. The differences in EO composition and dosage levels could lead to variability in carcass traits observed in chickens fed with EOs.

Excessive amounts of lipids lead to a chronic disease known as hyperlipidemia, which increases the risk of developing heart disease and stroke [41]. Our experiment indicated that the EOB supplementation increased HDL content and decreased TG, TC, and LDL-C concentrations in both the liver and serum of the birds. This decrease helps to avoid metabolic diseases. The supplementation of EOs in diet was reported to decrease plasma TG levels and enhance HDL concentrations, adjust the FA composition profile of the breast muscle, decrease drip loss, and improve the meat quality of the chickens [42]. Therefore, we speculated that the potential function of the EOB in improving meat quality might relate to its regulation of the lipid metabolism. Studies show that the administration of an EOB to birds reduces serum TC concentrations, which may be associated with the inhibitory effects of the EO compounds on the activity of 3-hydroxy-3-methylglutaryl coenzyme A reductase, a key enzyme in the regulatory pathway of TC synthesis [43].

The sensory and physical characteristics of meat, including color, texture, and flavor, are critical factors influencing consumer consumption choices [44]. We studied how the EOB supplementation influenced the breast meat quality, and found that the EOB_250_ treatment significantly elevated the yellow intensity of the breast fillets. Various factors regulate the color of broiler meat, including myoglobin and other sarcoplasmic proteins (including hemoglobin, cytochromes, and catalases), diet, age, pH, breed, sex, and management practices [45]. The yellowness of the breast fillets was found to be higher when the chickens were given an EOB of cinnamaldehyde, thymol, and carvacrol [46]. In another study, it was reported that the inclusion of thymol, cinnamaldehyde, and carvacrol showed an increase in yellowness, with or without curcumin added [47]. Besides the enhancement of color, the WHC was improved by the EOB supplementation, with decreased cooking and drip loss. In agreement with our findings, ref. [48] also reported that thyme EO in a broiler diet improved the WHC and minimized drip loss, thereby increasing tenderness. This improvement seems to be directly related to an increased oxidative defense system in the muscle [49]. Therefore, oxidation is a natural process that will affect meat quality by changing its pigments, fats, and proteins, resulting in a reduction in its shelf-life reduction [50]. Free radicals generated during lipid oxidation disrupt the membrane structure by targeting unsaturated fatty acids, lipoproteins, and other components within the phospholipid bilayer of cell membranes. This damage increases membrane permeability, compromising cellular integrity. Maintaining the structural integrity of cell membranes can help restrict the production of free radicals, reduce oxidative reactions, decrease the leakage of sarcoplasmic fluid, and enhance the WHC [51,52]. EOs contain bioactive compounds, including phenolics and terpenoids, which exhibit medicinal properties by reducing oxidative stress in broilers [52]. The increased levels of T-AOC, CAT, and GSH-Px suggested that improvement of the WHC, a factor contributing to lower cooking loss of the chicken meat for the EOB-supplemented group, also came from improvement of the muscle antioxidant capacity. Furthermore, higher muscle redox status and muscle water retention ability may be considered additional factors that could help to achieve improved meat quality results [53].

Texture profile analysis, a widely used method, uses a double compression cycle to mimic chewing, enabling the analysis of textural properties in the chicken meat [54]. This study also contributes to the understanding of the texture profile of the meat, specifically examining the effects of the EOB addition on cohesiveness and resilience. The EOB has the potential to improve meat tenderness, according to the findings. Similar findings [55] showed that the meat of chickens raised on diets with oregano or anise EOs had improved tenderness and juiciness. Two types of Mexican OEO in DW were tested in broiler chickens, and they improved meat quality by reducing cooking loss and increasing cohesiveness and resilience [27]. Physicochemical characteristics of meat, in general, can enhance the quality of chicken meat making it more palatable to consumers.

The activity of antioxidant defense enzymes in the body that reflects the physiological response of an organism to oxidative stress is tightly related to the general state of health [56]. Our results suggested that the EOB supplementation can improve antioxidant activity by increasing the T-SOD, CAT, GSH-Px, and T-AOC of the serum, liver, and breast muscle in broiler chickens. Several studies have reported that EOs can improve the oxidative stability of chicken tissues [57]. This study also found that the EOB supplementation lowered MDA levels, a marker of oxidative stress, in the liver and breast muscle tissues. MDA is a byproduct of the peroxidation of PUFAs in cells, and its excessive formation is driven by an increase in free radicals [58]. Consistent with our findings, similar studies have reported that the inclusion of aromatic plant extracts enhances GPx activity and reduces MDA levels [59,60]. Additionally, a balance was observed between the levels of reactive oxygen species and antioxidants in the chickens. The absence of secondary lipid peroxidation (MDA) and protein oxidation suggests that the broilers exhibited a robust antioxidant defense capacity, which effectively interrupted oxidative reactions and contained ROS. This indicates a potential protective effect of the EOB against oxidative damage. Nrf2 is a transcription factor that is a master regulator of cell antioxidant defense pathways. It induces cells to activate the expression of antioxidant response elements that regulate the expression of genes that control oxidative stress [61]. Broilers fed with EOB_250_ had a significantly higher mRNA level of *Nrf2*, demonstrating the stimulation of their intrinsic antioxidant system. Nrf2 upregulation suggests that the EOB supplementation may trigger a defensive mechanism against oxidative damage, thereby enhancing meat quality and stability. This antioxidant effect is combined by synergistic actions of T-SOD and CAT, in addition to GSH-Px, which work together to prevent reactive oxygen species (ROS) from inducing oxidative stress to muscle tissues. This study revealed that some antioxidants in the EOB entered the blood, remained in tissues, and protected the body from oxidative stress by building antioxidant enzymes.

Meat from PUFA-rich diets, especially n-3 PUFAs, provides health-promoting benefits. However, Our findings demonstrated that the PUFA content of muscle increased when the EOB was administered. The observed changes in fatty acid composition may be associated with the antioxidant properties of plant-derived compounds, such as flavonoids and terpenoids, and/or their ability to modify the gut microbial community [62,63], as well as the reduction of biohydrogenation of unsaturated fatty acids, which led to an increase in the PUFA/SFA rate [64]. Moreover, an upregulation of the expression of *FADS2* mRNA in birds supplied with the EOB was also detected, indicating that in birds receiving the EOB, the rise in PUFA levels was associated with upregulation of the *FADS2* pathway for PUFA synthesis. In the lipid metabolic pathway, *FADS2* is responsible for desaturating the FAs linoleate and alpha-linolenate into PUFAs [65]. Moreover, the birds that were supplemented with EOB_250_ and EOB_350_ had less SFA fractions, as shown by decreased C14:0, C16:0, and C18:0 contents. SFA is suggested to promote high levels of LDL-C and TC [66]. Consuming more SFAs in diets might lead to the hypercholesterolemic effects associated with coronary artery disease [67].

Meat health indices are valuable for analyzing the influence of a dietary FA profile on susceptibility to common chronic diseases. These indices are more cost-effective than lengthy laboratory procedures [68]. Whereas SFAs have been shown to have pro-inflammatory effects, EPA and DHA are considered anti-inflammatory [69]. The UI indicator for the level of unsaturation of FAs causing its structure, which is useful for assessing oxidative stability and suggesting possible oxidative protection approaches for livestock feed [70]. Also, the PI evaluates the stability of PUFAs in food, contributing to the prevention of oxidation processes [71]. In the relationship to health, NVI gives information about possible health consequences of the lipid profiles that positively correlate with the quality of the FAs [72]. More consistent with our findings, the elevated amounts of EPA + DHA, UI, and PI in the supplemented groups indicated a positive impact of the EOB supplementation on the health-promoting properties of chicken meat. IA and TI indices and h/H are good indicators of lipid health and nutritional quality. A lower IA and TI indicate a healthier fatty acid profile, which decreases the risk of platelet clumping and coronary diseases, while a high h/H ratio denotes a better quality of nutrition [73]. The EOB supplementation reduced TI and IA while elevating h/H in meat, indicating improved meat quality; it was demonstrated herein that the EOB_350_ and EOB_250_ levels of supplementation were the most effective treatment among the supported levels. Additionally, DFA, FLQ, and NVI serve as key markers of the overall health-promoting properties of the meat [36]. These results indicate that the EOB supplementation can improve meat quality, usually by modifying the FA profile in the breast meat, which is beneficial for consumer health.

To analyze the molecular mechanisms that led to alterations in the FA profile, we measured the expression of key genes associated with the liver lipid metabolism. A transcription factor, *SREBP-1c*, regulates genes associated with FA and TG synthesis, like *SCD, FAS*, and *ACC* [74]. In our study, the mRNA abundance level of *SREBP-1c* was reduced in the EOB-treated chickens compared to the CON, which was supported with lower levels of blood TG and TC. The activity of the EOB reduced blood TC and TG levels in experimental groups, which was related to decreased expression of *FAS* and *ACC*, especially in the EOB-treated groups. Another study reported similar findings, which revealed that carvacrol inhibits fat accumulation by downregulating *SREBP-1c* and *FAS*, thereby inhibiting lipogenesis and increasing *CPT1* expression to promote FA oxidation in high-fat diet-fed mice [75]. PPARγ is a nuclear receptor required for adipogenesis and acts by recruiting *C/EBPα* to generate a transcriptional complex regulating adipocyte differentiation and lipid storage [76]. In the present study, *PPARγ* mRNA expression level was higher in the CON group than the EOB_250_. This finding follows other studies [77] that cinnamaldehyde inhibits the development of adipocytes and modulates adipose tissue metabolism by downregulating *PPARγ* and *C/EBPα*. This regulation leads to the prevention of adipocyte differentiation and fat accumulation. Cinnamaldehyde also restricted the expression of *SREBP-1c* and *FAS*, which can also inhibit FA synthesis and lipid storage.

To further understand the impact of the EOB on lipid metabolism, we assessed the expression level of enzymes involved in FA catabolism in liver tissue. *PPARα* regulates numerous metabolic processes that facilitate cellular uptake of FA and metabolism via FA *β*-oxidation, ultimately promoting cellular respiration [78], including expression of *CPT1*, which is responsible for FA transport into mitochondria for *β*-oxidation. ACOX1 is another important enzyme for FA catabolism in the peroxisome, which is considered a rate-limiting step during peroxisomal β-oxidation [78]. In this study, supplementation of the EOB increased the mRNA expression of *PPARα*, *ACOX1*, and *CPT1* in liver tissue compared to the CON. These results are in agreement with previous findings, which showed that cinnamon can upregulate PPARα gene expression in liver tissue while decreasing serum and liver TC levels as well as serum TG. Generally, these findings suggested that the EOB modulates FA β-oxidation, at least in part by activating the *PPARα* pathway, leading to enhanced FA β-oxidation [79]. To summarize, the EOB supplementation increased ABW, FCR, and breast weight compared to the CON, which may have a major economic impact on the industry.

## 5. Conclusions

This investigation revealed that supplementing drinking water with EOB_250_ and EOB_350_ improved breast fillet yellowness, WHC, and unsaturated fatty acid content while reducing drip loss and cooking loss. Additionally, these groups demonstrated enhanced lipid quality indices and antioxidant activity in the breast muscle. Moreover, EOB_250_ and EOB_350_ were found to regulate liver lipid metabolism by downregulating the expression of genes associated with fatty acid synthesis and upregulating those involved in fatty acid oxidation. These results indicate that EOB supplementation enhances the overall meat quality of broilers, making it more nutritionally beneficial. In conclusion, this study suggests that supplementing broiler drinking water with 250 mg/L of the EOB holds significant potential as an effective alternative water additive for the broiler industry.

## Figures and Tables

**Table 1:** Ingredients and nutrient composition of basal diets.

Items	1−21 d	22−42 d
Ingredient (%)		
Corn	58.36	64.22
Soybean meal	31.25	27.08
Soybean oil	4	3
Corn gluten meal	2.8	2.5
Limestone	1.29	1.42
Dicalcium phosphate	1.49	1.04
L-Lysine	0.03	0.03
DL-Methionine	0.23	0.16
Sodium chloride	0.3	0.3
Premix ^1^	0.25	0.25
Total	100	100
Calculated composition		
Metabolic energy MJ/kg	12.65	12.67
Crude protein, %	21	19
Crude ash, %	6	8
Calcium, %	0.9	1.1
Total phosphorus, %	0.4	0.45
Methionine, %	1.1	1.04
Lysine, %	0.52	0.9

^1^ Premix provided per kilogram of diet: vitamin A (trans-retinyl acetate), 10,000 IU; vitamin D3 (cholecalciferol), 3000 IU; vitamin E (all-rac α-tocopherol), 30 IU; menadione, 1.3 mg; thiamin, 2.2 mg; riboflavin, 8 mg; nicotinamide, 40 mg; choline chloride, 600 mg; calcium pantothenate, 10 mg; pyridoxine. HCl, 4 mg; biotin, 0.04 mg; folicacid,1 mg; vitamin B12 (cobalamin), 0.013 mg; Fe (from ferrous sulfate), 80 mg; Cu (from copper sulfate), 8.0 mg; Mn (from manganese sulfate), 110 mg; Zn (from zinc oxide), 60 mg; I (from calcium iodate), 1.1 mg; Se (from sodium selenite), 0.3 mg.

**Table 2:** Primers used in the present study.

Gene	Primer Sequence (5′–3′)	Accession No.
*β-actin*	F: ACCGGACTGTTACCAACACC	NM 205518.1
	R: CCTGAGTCAAGCGCCAAAAG	
*CAT*	F: GGTTCGGTGGGGTTGTCTTT	NM_0,010,31215.1
	R: CACCAGTGGTCAAGGCATCT	
*SOD*	F: CCGGCTTGTCTGATGGAGAT	NM_205,064.1
	R: TGCATCTTTTGGTCCACCGT	
*GSH*	F: GACCAACCCGCAGTACATCA	NM_0,012,77853.1
	R: GAGGTGCGGGCTTTCCTTTA	
*NRF2*	F: GATGTCACCCTGCCCTTAG	NM_205,117.1
	R: CTGCCACCATGTTATTCC	
*SREBP-1c*	F: GCCCTCTGTGCCTTTGTCTTC	XM_046927256.1
	R: ACTCAGCCATGATGCTTCTTC	
*ACC*	F: TTGTGGCACAGAAGAGGGAA	NM_205505.1
	R: GTTGGCACATGGAATGGCAG	
*FAS*	F: AGAGGCTTTGAAGCTCGGAC	NM_205155.2
	R: GGTGCCTGAATACTTGGGCT	
*FADS2*	F: AATTGAGCACCACCTGTTCC	NM_001160428.2
	R: TGGCACATAACGACTTCACC	
*SCD*	F: CATGGGCCATTCTGTGCTT	NP_990221.2
	R: GGCCATGGAGTTTGCAATAG	
*PPARγ*	F: CCAAGGCAGCGGCAAAATAA	NM001001460
	R: GTGCCCATAAATGATGGCCTAA	
*C/EBPα*	F: GACATCTGCGAGAACGAGCA	NM001031459
	R: GCATGCCGTGGAAATCGAAA	
*PPARα*	F: AGTAAGCTCTCAGAAACTTTGTTG	NM_001001464.1
	R: AAGGTTGAAACAGAAGCCGC	
*ACOX1*	F: GCCAGGTGGACTTGGAAAGA	NM_001012578
	R: GCTGCCGTATAGGAACAATGAAG	
*CPT1*	F: ACAGCGAATGAAAGCAGGGT	NM_0,010,31215.1
	R: CACCAGTGGTCAAGGCATCT	
*LPL*	F: CCGATCCCGAAGCTGAGATG	NM205282
	R: ACATTCCTGTCACCGTCCAC	
*FABP*	F: AGAAGGCCAAGTGTATTGTTAACAT	NM_204192.3
	R: GTGATGGTGTCTCCGTTGAGTTC	
*FATB1*	F: CTACACTTCGGGTACGACGG	NM_001398142.1
	R: GTAGAGCGGAAGGCAGTTGT	

**Table 3:** Effects of essential oils blend on growth performance of broilers.

Items ^1^	Essential Oils Blend (EOB mg/L)				SEM ^2^	*p*-Value
	CON	EOB_150_	EOB_250_	EOB_350_		
D 1−21						
IBW (g)	46.13	45.91	46.42	46.74	0.235	0.647
ABW g/bird	982.30	976.35	989.50	998.25	8.130	0.814
ADG g/bird/d	44.57	44.30	44.90	45.30	0.387	0.832
ADFI g/bird/d	59.10	58.28	58.14	59.05	0.400	0.779
FCR	1.32	1.31	1.29	1.30	0.011	0.833
D 22−42						
ADG g/bird/d	87.81	88.06	94.41	90.79	1.323	0.287
ADFI g/bird/d	146.22	153.22	154.54	155.89	1.619	0.140
FCR	1.67	1.74	1.64	1.72	0.023	0.477
D 1−42						
ABW g/bird	2826.44	2825.66	2973.19	2904.9	30.815	0.291
ADG g/bird/d	66.19	66.18	69.68	68.05	0.732	0.294
ADFI g/bird/d	102.66	105.75	106.42	107.47	0.873	0.235
FCR	1.55	1.59	1.53	1.58	0.015	0.480

^1^ IBW: initial weight; ABW: average body weight; ADG: average daily gain; ADFI: average daily feed intake; FCR: feed conversion ratio. ^2^ SEM: standard error of the mean; CON = control drinking water (without EOB); EOB_150_ = DW + 150 mg/L EOB; EOB_250_ = DW + 250 mg/L EOB; EOB_350_ = DW + 350 mg/L EOB.

**Table 4:** Effect of essential oils blend on the carcass characteristics of broilers.

Items	Essential Oils Blend (EOB mg/L)				SEM ^1^	*p*-Value
	CON	EOB_150_	EOB_250_	EOB_350_		
Liver index (%)	1.97	1.92	2.04	2.13	0.065	0.686
Spleen index (%)	0.10	0.10	0.10	0.10	0.004	0.909
Bursa of Fabricius index (%)	0.12	0.13	0.12	0.13	0.007	0.863
Breast index (%)	22.38 ^b^	23.97 ^ab^	24.77 ^a^	23.73 ^ab^	0.304	0.037
Abdominal fat index (%)	1.47	1.34	1.33	1.34	0.082	0.922
Semi-evisceration index (%)	84.97	86.24	85.41	88.53	0.611	0.166
Fully-evisceration index (%)	71.90	72.93	72.64	72.65	0.219	0.396

^a,b^ Means in the same row with different superscripts are significantly different (*p* < 0.05). ^1^ SEM: standard error of the mean. CON = control drinking water (without EOB); EOB_150_ = DW + 150 mg/L EOB; EOB_250_ = DW + 250 mg/L EOB; EOB_350_ = DW + 350 mg/L EOB.

**Table 5:** Effect of essential oils blend on meat quality of chicken breast muscle.

Items ^1^	Essential Oils Blend (EOB mg/L)				SEM ^2^	*p*-Value
	CON	EOB_150_	EOB_250_	EOB_350_		
pH value	6.01	6.22	6.20	6.21	0.094	0.846
WHC	79.52 ^b^	81.99 ^a^	82.77 ^a^	82.39 ^a^	0.450	0.037
Drip loss (%)	1.97 ^a^	1.87 ^ab^	1.54 ^b^	1.53 ^b^	0.070	0.027
Cooking loss (%)	18.35 ^a^	16.49 ^ab^	16.39 ^ab^	15.07 ^b^	0.420	0.042
Shear force	21.83	20.01	19.79	20.01	0.980	0.888
L*	38.39	41.36	39.44	40.80	0.630	0.346
a*	1.02	1.34	1.38	1.25	0.110	0.677
b*	3.51 ^c^	3.88 ^bc^	5.08 ^a^	4.77 ^ab^	0.187	0.004
ΔE	54.70	51.78	53.77	52.37	0.628	0.355
Hue	4.10	3.99	5.20	4.53	0.479	0.822
Chroma	3.67 ^c^	4.16 ^bc^	5.30 ^a^	4.95 ^ab^	0.193	0.006
BI	11.41 ^b^	12.06 ^b^	15.93 ^a^	14.35 ^ab^	0.651	0.043

^a–c^ Means in the same row with different superscripts are significantly different (*p* < 0.05). ^1^ WHC: water holding capacity; L*: lightness; a*: redness; b*: yellowness; ΔE: total color change; Hue: Hue angle; Chroma: saturation index; BI: browning index. ^2^ SEM: standard error of the mean. CON = control drinking water (without EOB); EOB_150_ = DW + 150 mg/L EOB; EOB_250_ = DW + 250 mg/L EOB; EOB_350_ = DW + 350 mg/L EOB.

**Table 6:** Effect of essential oils blend on fatty acid profile from breast muscle in broilers.

Items ^1^	Essential Oils Blend (EOB mg/L)				SEM ^2^	*p*-Value
	CON	EOB_150_	EOB_250_	EOB_350_		
C12:0	0.03 ^a^	0.03 ^a^	0.023 ^b^	0.025 ^b^	0.001	0.020
C14:0	0.72	0.70	0.69	0.69	0.008	0.506
C16:0	24.96	24.81	23.56	23.90	0.274	0.196
C18:0	8.44 ^a^	7.51 ^ab^	7.10 ^b^	7.17 ^b^	0.189	0.034
C16:1	3.96	4.28	4.62	4.20	0.183	0.682
C18:1n9	39.37	39.63	42.05	41.63	0.507	0.138
C18:2n6	14.95 ^ab^	14.85 ^b^	15.94 ^a^	15.91 ^a^	0.182	0.035
C18:3n3	2.21 ^b^	2.48 ^ab^	2.86 ^a^	2.89 ^a^	0.088	0.007
C18:3n6	0.29	0.34	0.37	0.35	0.012	0.166
C20:4n6	4.29 ^b^	4.62 ^ab^	5.20 ^a^	5.10 ^a^	0.128	0.029
C20:5n3	0.21	0.22	0.25	0.26	0.007	0.056
C22:4n6	0.68 ^b^	0.69 ^b^	0.77 ^a^	0.72 ^ab^	0.010	0.004
C22:6n3	2.44	2.54	2.92	2.62	0.067	0.053
SFA	34.16 ^a^	33.06 ^ab^	31.38 ^b^	31.78 ^b^	0.383	0.030
MUFA	43.34	43.92	46.67	45.84	0.612	0.175
PUFA	25.11 ^b^	25.75 ^b^	28.33 ^a^	27.87 ^a^	0.325	0.001
n-6 PUFA	20.24 ^b^	20.51 ^b^	22.28 ^a^	22.10 ^a^	0.257	0.001
n-3 PUFA	4.87 ^c^	5.24 ^bc^	6.05 ^a^	5.77 ^ab^	0.131	0.001

^a–c^ Means in the same row with different superscripts are significantly different (*p* < 0.05). ^1^ Lauric acid (C12:0); Myristic acid (C14:0); Palmitic acid (C16:0); Stearic acid (C18:0); Palmitoleic acid (C16:1); Oleic acid (C18:1n9); Linoleic acid (LA, C18:2n6c); α-Linolenic acid (ALA, C18:3n3); γ-linoleic acid (C18:3n6); Arachidonic acid (AA, C20:4n6); Eicosapentaenoic acid (EPA, C20:5n3); Docosatetraenoic acid (C22:4n6); Docosahexaenoic acid (DHA, C22:6n3); SFA: Saturated fatty acid; MUFA: Monounsaturated fatty acid; PUFA: Polyunsaturated fatty acid. SFA percentage is the sum of C12:0, C14:0, C16:0, and C18:0; MUFA percentage was calculated as the sum of C16:1and C18:1n-9; PUFA percentage was calculated as the sum of C18:2n6c, C18:3n3, C18:3n6, C20:4n6, C20:5n3, C22:4n6, and C22:6n3. ^2^ SEM: standard error of the mean. CON = control drinking water (without EOB); EOB_150_ = DW + 150 mg/L EOB; EOB_250_ = DW + 250 mg/L EOB; EOB_350_ = DW + 350 mg/L EOB.

**Table 7:** Impact of essential oils blend on lipid quality indexes in breast muscle.

Items ^1^	Essential Oils Blend (EOB mg/L)				SEM ^2^	*p*-Value
	CON	EOB_150_	EOB_250_	EOB_350_		
Qualitative						
n-6/n-3	4.17	3.95	3.72	3.83	0.087	0.303
PUFA/SFA	0.73 ^b^	0.78 ^b^	0.90 ^a^	0.87 ^a^	0.018	0.001
LA/ALA	6.78	6.04	5.73	5.55	0.177	0.059
EPA + DHA	2.65 ^b^	2.76 ^b^	3.18 ^a^	2.88 ^ab^	0.070	0.036
UI	116.47 ^b^	119.74 ^b^	131.02 ^a^	127.79 ^a^	1.409	0.001
Nutritional						
NVI	1.91	1.90	2.09	2.04	0.029	0.051
TI	0.73 ^a^	0.68 ^a^	0.59 ^b^	0.61 ^b^	0.015	0.001
IA	0.40 ^a^	0.39 ^a^	0.35 ^b^	0.36 ^b^	0.006	0.002
h/H	2.50 ^b^	2.56 ^b^	2.91 ^a^	2.82 ^a^	0.048	0.001
HPI	2.45 ^b^	2.52 ^b^	2.85 ^a^	2.76 ^a^	0.048	0.002
FLQ	7.78 ^b^	8.42 ^b^	10.18 ^a^	9.10 ^ab^	0.276	0.007
PI	61.84 ^b^	64.55 ^b^	72.45 ^a^	69.45 ^a^	1.037	0.001
IB	19.77	20.81	20.20	19.78	0.634	0.941
DFA	76.90 ^b^	77.20 ^b^	82.12 ^a^	80.88 ^a^	0.670	0.003
NR	0.47 ^a^	0.47 ^a^	0.41 ^b^	0.42 ^b^	0.007	0.008
Metabolic						
Elongase	33.93	30.34	30.15	30.06	0.741	0.187
Thioesterase	3444.08	3553.36	3392.01	3453.94	44.544	0.662
∆9-Desaturase (16)	13.64	14.66	16.35	14.96	0.580	0.449
∆9-Desaturase (18)	82.28 ^b^	83.96 ^ab^	85.54 ^a^	85.30 ^a^	0.458	0.033
∆9-Desaturase(16 + 18)	56.43 ^b^	57.52 ^ab^	60.34 ^a^	59.60 ^a^	0.539	0.025
Activity index	2.19	2.11	2.14	2.01	0.035	0.312

^a,b^ Means in the same row with different superscripts are significantly different (*p* < 0.05). ^1^ PUFA/SFA: Σ Polyunsaturated Fatty Acid/Σ Saturated Fatty Acid; LA/ALA: Linoleic Acid/α-Linolenic Acid; EPA + DHA: Eicosapentaenoic Acid and Docosahexaenoic Acid; UI: Unsaturation index; NVI: Nutrition Value Index; TI: thrombosis index; IA: Index of Atherogenicity; h/H: Hypocholesterolemic/Hypercholesterolemic; HPI: Health-Promoting Index; FLQ: Flesh Lipid Quality; PI: Peroxidation trend index; IB: Inflammatory Biomarker; DFA: Dietary fatty acids; NR: Nutritional ratio; ^2^ SEM: standard error of the mean. CON = control drinking water (without EOB); EOB_150_ = DW + 150 mg/L EOB; EOB_250_ = DW + 250 mg/L EOB; EOB_350_ = DW + 350 mg/L EOB.

## Data Availability

The data presented in this study are available on request from the corresponding author.
